# Supplementary material for: Autistic adults’ experiences of accessing and receiving mental health care and their priorities for improvements: A qualitative study
Source: PLOS Ment Health. 2026 May 19;3(5):e0000581. doi: 10.1371/journal.pmen.0000581 (PMC13186342; doi:10.1371/journal.pmen.0000581)
Supplement: Attachment — Submitted filename: Response to Reviewers.docx [file pmen.0000581.s004.docx]

**Manuscript number: PMEN-D-25-00324**

**Reviewer 1**

Hello, and thank you for allowing me to read this important study. Your results align well with other similar studies, both in mental health and somatic health. Great that you have involved autistic people in the development of the interview guide. I want to commend you for that. I think there are some revisions you could make before the manuscript is ready for publication. My comments are:

1. Explain why your broader focus is important (in the rationale section).

**Author response:** Thank you. Added before Aims (page 4, lines 93-96):

***”A key strength of the current study is its broader focus on autistic adults’ experiences across general mental health services, enabling identification of common barriers and priorities for improvement that apply across diverse contexts and providing insights that are more widely applicable to policy and practice in adult mental health care”***

2. In the method section: provide more details regarding recruitment. How many people expressed interest in participating in the study? Were you able to include all of them? Did anyone drop out after expressing interest, and if so, why?

**Author response:** Thank you. Added in recruitment and consent (page 6-7, lines 137-148):

***”The study was advertised on the National Survivor User Network’s (NSUN) weekly bulletin to their network of over 5,000. This generated approximately forty expressions of interest. There were plans to recruit through other channels, but we received a very positive response from NSUN — a nationwide network that does not focus on any specific mental health condition or demographic group, making it a strong route to recruiting a diverse sample. Recruitment remained open while interviews were conducted, and demographic information (age, gender, ethnicity, sexual orientation, and types of mental health service use) was collected only after consent during the interviews themselves. Once the target sample size was reached and the consented sample demonstrated good demographic variability across these characteristics, providing a rich data set with a full range of perspectives, recruitment was closed. It was therefore decided to recruit solely from this group using a pragmatic approach, prioritising individuals who confirmed their eligibility through initial email discussions with the first author. No participants dropped out after interviews were arranged.”***

3. Specify in the inclusion criteria that you also include self-identified autistic people.

**Author response:** Thank you. Inclusion criteria described as follows (page 5, lines 119-120):

**“We aimed to recruit between 12-15 participants who were over 18 years of age, had received care from mental health services in England and who identified as autistic (including those with a formal diagnosis and those who self-identified as autistic)”.**

4. How was the interview guide pilot-tested? For example, how many pilot-interviews were there and with whom? Did the pilot interviews result in any changes?

**Author response:** Thank you. Pilot test described as follows (page 7, lines 164-170):

***”The interview guide was pilot tested through a practice interview conducted by the first author with Nafiso Ahmed (a co-author and research fellow on the team, who is also the parent of an autistic child). This pilot interview helped assess the clarity, flow and length of the questions. Minor adjustments were made to wording and the addition of prompts to improve participant comprehension and encourage elaboration. The topic guide was further reviewed following the first two interviews to ensure that the questions were clear, acceptable and relevant to the objectives.”***

5. Reflexivity: Include your theoretical standpoint. Do you see data from a biomedical (traditional) perspective or a neurodiversity perspective? This is not crystal clear. For example, you use both “camouflage” (biomed) and “masking” (neudiv.) in the result section.

**Author response:** Thank you. Added to reflexivity statement (page 10, lines 222-224):

***“Our research was informed by a neurodiversity-affirming perspective where autism is viewed as natural human variation rather than a deficit. This perspective likely shaped our focus on participants’ lived experiences, while giving less emphasis to biomedical or deficit-based framings of autism.”***

**Use of ’camouflage’ removed throughout**

6. In the discussion, you could include a broader perspective as well. For example, discuss that similar challenges exist in all areas of healthcare, not just in mental health. For example: Hedlund Å, Andersson A, Lindberg M, Jordal M. Experiences and perceptions of physical healthcare among adult autistic patients: A scoping review. *Int J Nurs Stud Adv*. 2025;9:100366. Published 2025 Jun 10. doi:10.1016/j.ijnsa.2025.100366

**Author response:** Thank you. Added to discussion section (pages 25-26, lines 552-558):

***“Similar challenges to those reported in mental health services have also been documented across other areas of healthcare for autistic adults. A recent scoping review of experiences and perceptions of physical healthcare among autistic patients identified common barriers including communication difficulties, sensory sensitivities, inflexible systems and lack of clinical knowledge of autism, often resulting in unmet needs, negative perceptions of care and avoidance of services [33]. These similarities suggest systemic issues affect autistic adults across healthcare domains which reinforces the urgency of co-produced improvements beyond mental health services alone.”***

7. Expand the discussion regarding the double empathy problem, i.e., that it is not just about misunderstandings between different people, but specifically between autistic and non-autistic people, and explain how healthcare professionals can address the double empathy problem.

**Author response**: Thank you. Added to findings in context (page 25, lines 541-545):

**”... specifically between autistic and non-autistic people due to differences in communication styles, sensory experiences and ways of interpreting the world. Healthcare professionals can help address the double empathy problem by adopting clear and direct communication, allowing extra processing time and actively seeking clarification rather than assuming understanding.”**

**Reviewer 2**
Introduction

1. The writing is clear, but this section felt overly short, especially for a qualitative manuscript. Indeed, some key references to the literature are missing that struck me as relevant as soon as I read the Abstract (some examples below). Overall, I feel this section is a little under-developed.
   Crane, L., Adams, F., Harper, G., Welch, J., & Pellicano, E. (2019). ‘Something needs to change’: Mental health experiences of young autistic adults in England. Autism, 23(2), 477-493.
   Cooper, K., Loades, M. E., & Russell, A. (2018). Adapting psychological therapies for autism. Research in autism spectrum disorders, 45, 43-50.

**Author response:** Thank you. Significant additions made to introduction section (page 2-5, lines 50-102).

Method

2. NIHR acronym needs explaining for non-UK readers.

**Author response:** Thank you. Acronym explained (page 5, lines 104-105):

3. 'Lived Experience Researcher group' should also be explained - as this is within my field I will assume this means autistic people who are researchers and/or academics, but this needs spelling out for the reader as PLOS is a broad-interest journal.

**Author response:** Thank you. Lived Experience Researcher group explained (page 5, lines 108-109)

***...‘a group of autistic adults with lived experience of mental health difficulties who work as paid researchers within the unit and are regularly consulted regarding study questions, design, interpretation of findings and dissemination across studies.’***

4. In the Participants section, there is some discussion of the sampling strategy. But I wonder reading this whether the authors also considered the diversity of their sample (gender, age, ethnicity, SES, etc), and how this fed into their sampling and recruitment here? (I see in the Results section that a diverse sample was achieved, but this also needs discussing in the Participants section in terms of your efforts here).

**Author response:** Thank you for your helpful comment. Yes, that’s correct. We aimed to recruit a diverse sample and adopted a pragmatic approach. We agree that further information explaining this would be helpful in our methods section, so we have revised page 6-7 (lines 138-148) to include below:

***“There were plans to recruit through other channels, but we received a very positive response from NSUN — a nationwide network that does not focus on any specific mental health condition or demographic group, making it a strong route to recruiting a diverse sample. Recruitment remained open while interviews were conducted, and demographic information (age, gender, ethnicity, sexual orientation, and types of mental health service use) was collected only after consent during the interviews themselves. Once the target sample size was reached and the consented sample demonstrated good demographic variability across these characteristics, providing a rich data set with a full range of perspectives, recruitment was closed. It was therefore decided to recruit solely from this group using a pragmatic approach, prioritising individuals who confirmed their eligibility through initial email discussions with the first author. No participants dropped out after interviews were arranged.”***

5. 'Experience of accessing and receiving mental health care' needs defining (end of P.4) - this is a huge range of services so it needs some definition. Could this be seeing a psychiatrist for medication? Attending a service for talking therapies? Other? etc.

**Author response:** Thank you for this feedback. We agree, and have included a defintion on page 6 (lines 122-124):

**Defined as ’This included any contact with specialist NHS mental health services such as community mental health teams, early intervention in psychosis services, home treatment teams, outpatient talking therapies or inpatient psychiatric care’**

6. I think I am correct in understanding that recruitment only happened via NSUN? If so, what are the implications of this for your sample and their possible experiences? I can see that the authors state they received sufficient numbers from this network, but I think this curtails the diversity of experiences you would have been able to achieve with a broader recruitment strategy (you could for example have restricted numbers recruited from expressions of interest from that network, and still gone ahead with recruiting via other networks too). This network may, for example, be more likely to have experienced trauma, etc. This needs addressing here and/or in the Discussion.

**Author response:** Thank you for this feedback. We have added to strenghts and limitations (page 26-27, lines 577-581):

***“Recruiting exclusively through NSUN enabled direct access to autistic adults with lived experience of mental health difficulties, avoided clinician gatekeeping and generated a diverse sample. However, recruiting from this single source may have excluded individuals disengaged from service user networks, possibly skewing the sample towards more critical or reflective voices engaged in lived-experience advocacy while excluding those less connected to such communities.*”**

7. More information is needed in the Recruitment and Consent section about the information participants received - what did this tell them about the study, its aims, etc?

**Author response:** Thank you. We have added under recruitment and consent (page 7, lines 150-155):

***”This sheet explained the purpose of the study (to explore autistic adults’ experiences of mental health care in England and their priorities for service improvements), what participation would involve, the voluntary nature of participation, confidentiality procedures and possible emotional risks and available support. It also provided an overview of the main topics to be discussed. The full interview questions were not shared in advance to allow for a flexible, semi-structured conversation that could adapt to each participant’s communication style and priorities.”***

8. It would also be helpful to specify whether any specific people or groups were or would have been excluded (e.g., individuals with an Intellectual Disability), etc.

**Author response:** Thank you. We have added under ’Participants’ (page 6, lines 125-127):

**”Individuals with co-occurring intellectual disabilities were not specifically targeted in recruitment and were excluded as the study materials and interview format were designed for autistic adults without intellectual disability.”**

Also acknowledged as a limitation (page 26, lines 573-577):

**”The topic guide and recruitment materials targeted autistic adults without intellectual disabilities, limiting relevance for those with such co-occurrences. While the study information and data collection materials were designed to be accessible, someone with a significant intellectual disability would be unlikely to be able to give informed consent on the basis of them alone.”**

9. It is unclear where the interviews were conducted. If remotely, this needs to be specified, along with details of method/s and numbers using each (video call, telephone, etc).

**Author response:** Thank you. We have added under data collection and management (page 8, lines 179-180):

**”All 13 interviews were conducted remotely via Microsoft teams online video calls (no telephone or in-person interviews were used).”**

10. Were participants provided with the interview questions or an idea of the topics in advance? If not, why not?
    **Author response:** Thank you. Added to recruitment and consent (page 7, lines 154-155):

**“The full interview questions were not shared in advance to allow for a flexible, semi-structured conversation that could adapt to each participant’s communication style and priorities.”**

11. End of P.6: "implementing feedback from a Lived Experience Researcher on the relevance of the identified themes to the target population" needs explaining further, with examples. Generally, references to the roles of the Lived Experience Researcher/s are lacking in sufficient detail throughout the manuscript.

**Author response:** Thank you. Added to method section (page 5, lines 109-113):

**”Two Lived Experience Researchers provided detailed written and verbal feedback on early drafts of the interview guide, suggesting rephrasing of some questions to make them clearer and more relevant to autistic service users and proposing additional prompts. They also reviewed emerging themes during analysis to ensure the interpretation remained grounded in autistic lived experience.”**

Added to ‘Analysis’ section (page 8-9, lines 195-200):

**“At this stage, a draft thematic table was shared with an autistic Lived Experience Researcher. She provided detailed written feedback confirming their relevance and suggesting minor refinements to better reflect autistic experiences such as consideration of communication barriers in accessing services and the role of special interests. This feedback informed refinements to theme naming and framing to ensure the final themes better reflected autistic lived experience.”**

Results

12. Good to see a relatively diverse sample. Layout of results and table of themes is clear and as expected based on prior literature.
    **Author response:** Thank you!
13. Themes/subthemes on P.14 relevant for a brief discussion about 'diagnostic overshadowing', which the authors may wish to include. Similarly, 'therapeutic alliance' is relevant to the themes discussed on P.16.

**Author response:** Thank you. Edit to results section: iv) Lack of consideration of autism alongside mental health condition(s) (page 16, lines 348-352)

**Some participants described experiences consistent with diagnostic overshadowing, where services failed to adequately consider the interplay between their autism and co-occurring mental health conditions, resulting in ineffective and impersonal interventions.**

**“It’s either, you get seen as kind of an autistic person and that’s the primary thing and everything stems from that […] or you get treated as like a mentally ill person and everything else stems from that.” (P6)**

And edit to i) The importance of connection and continuity with familiar people in mental health care (page 18, lines 387-389):

**”Many participants expressed concerns about services where they were offered support by ever-changing unfamiliar professionals and described how this hindered the development of a strong therapeutic alliance and thus the success of their treatment: ”**

14. I think the Results section could be further improved by including either another table or an infographic of sorts to highlight the recommendations coming from your findings.

**Author response:** Thank you. Table added to results section (page 22-23, lines 494-495):
**Table 3. Participant priorities for improving mental health services for autistic adults**

| Priority | Key suggestions | Illustrative quote |
| --- | --- | --- |
| Adaptability and person-centred care | Flexible, individually tailored treatment plans; ask patients what works for them | “Not everyone’s going to find the same things useful… what works for you? What doesn’t?” (P1) |
| Greater consideration of autism alongside mental health conditions | Treat autism and mental health conditions as intertwined rather than one overshadowing the other | “My autism plays a really big part in my eating disorder.” (P10) |
| Improved knowledge of autism among professionals | Mandatory autism training co-produce with autistic people | “Mental health professionals really need to be trained in autism… developed by people with autism… real co-production.” (P2) |
| Simple communication adjustments | Direct questions, avoid open-ended questions, allow preparation time, no forced eye contact | “It broke it down so there isn’t that just ‘Right, so how are you doing?’ kind of big open question.” (P5) |

| Earlier diagnosis of autism | Increased investment in timely diagnosis (especially for females) | “More investment in… diagnosis for children because with females they don’t even start to recognise it until a certain age.” (P11) |
| --- | --- | --- |
| Awareness of sensory needs | Offer ear defenders, quiet spaces, sensory rooms or simple enquiries about sensory triggers | “A small adjustment might even be having ear defenders… ‘Are you finding the noise tough?’” (P9) |

Discussion

15. The initial part of this section is somewhat under-developed, especially for a qualitative paper.

**Author response:** Thank you. Significant additions made discussion (page 23-26, lines 497-558.

16. Your sample includes a fairly high number of people who've been psychiatric inpatients - the implications of this should be discussed.
    **Author response:** Thank you. Addition to strenghts and limitations (page 27, lines 586-588):

**“A relatively high proportion of the sample (60%) had experienced psychiatric inpatient services which may reflect greater representation of negative or crisis-related experiences”**

17. Some discussion about the relative pros & cons of including a self-diagnosed interviewee should be discussed.

**Author response:** Thank you. Added to pros and cons section of discussion (page 27, lines 588-591):

**”The inclusion of one self-identified (rather than formally diagnosed) autistic participant increased the diversity of perspectives and aligned with principles that value self-identification; however, it limits direct comparability with studies that required formal diagnosis.”**
