# Supplementary material for: Autistic adults’ experiences of accessing and receiving mental health care and their priorities for improvements: A qualitative study
Source: PLOS Ment Health. 2026 May 19;3(5):e0000581. doi: 10.1371/journal.pmen.0000581 (PMC13186342; doi:10.1371/journal.pmen.0000581)
Supplement: Attachment — Submitted filename: Response to Reviewers 24.2.docx [file pmen.0000581.s005.docx]

**Reviewer comments**

1. Inclusion of a self-identified participant

**Author response:**

**Thank you. The paragraph in the Strengths and limitations section has been revised. The reference to ‘comparability’ has been removed (page 27, lines 592-594):**

**‘The inclusion of one self-identified (rather than formally diagnosed) autistic participant increased the diversity of perspectives and aligned with principles that value self-identification [36]; however, self-identification carries an inherent risk that some experiences described may relate to other conditions or explanations other than autism.’**

2. Support needs heterogeneity

**Author response:**

**Thank you. A statement has been added to the Strengths and limitations section alongside the intellectual disability exclusion limitation (page 26, lines 577-579):**

**‘The study also did not assess or differentiate participants according to levels of support needs, an important source of heterogeneity within autistic populations that may influence experiences of accessing and receiving mental health care.’**

**Administrative requirements.**

- Competing interest statement below:

*I have read the journal's policy and the authors of this manuscript have the following competing interests: NA, BLE and SJ currently work at the same universitye, UCL, as the editor chief of PLOS Mental Health, RB. All other authors declare no competing interests.*

- Funding information for this study below:

*This study was conducted as the dissertation project for the first author’s MSc studies at University College London. Reimbursement to participants and lived experience researchers, as well as transcription costs, were supported by funds from the UCL course budget available to all projects within the MSc cohort. UCL had no role in the study design, data collection and analysis, decision to publish, or preparation of the manuscript.*

- Data availability statement:

We have sent an email to the journal formally requesting for exemption regarding data availability due to ethical constraints.
